# LncRNA–protein interactions: molecular switches for the precise regulation of innate immunity

**DOI:** 10.3389/fimmu.2026.1994058

**Published:** 2026-10-06

**Authors:** Chenhao Yang, Yanxia Feng, Chengfang Li, Ting He, Linglong Xu, Zilin Qiao, Jiamin Wang, Zhenbin Liu

**Affiliations:** 1 School of Bioengineering, Northwest Minzu University, Lanzhou, China; 2 Ministry of Education Engineering Research Center for Key Technologies and Industrialization of Cell-Based Vaccines, Northwest Minzu University, Lanzhou, China; 3 Gansu Animal Cell Technology Innovation Center, Northwest Minzu University, Lanzhou, China; 4 State Ethnic Affairs Commission Key Laboratory of Bioengineering and Technology, Northwest Minzu University, Lanzhou, China

**Keywords:** antiviral immunity, innate immunity, lncRNA-protein interaction, long non-coding RNA, protein regulation

## Abstract

Long non-coding RNAs (lncRNAs) have emerged as important regulators of innate immunity, acting across multiple molecular layers to shape antiviral and inflammatory responses. Among their diverse functions, protein-centered regulation provides a particularly versatile means of controlling immune signaling by altering the functional state, interactions, localization, or turnover of proteins without necessarily changing their transcription. In this review, we synthesize current evidence for lncRNA-mediated protein regulation in innate immunity and propose a protein-centered framework that connects molecular regulatory mechanisms with their functional and immunological consequences. At the mechanistic level, lncRNA–protein interactions can modulate post-translational modifications, alter protein conformations, and facilitate or disrupt protein–protein interactions. These regulatory modes, in turn, affect four major properties of immune proteins: protein stability, subcellular localization, molecular activity, and protein–protein interaction-dependent signal transmission, ultimately reshaping innate immune responses. Across these mechanisms, several recurrent principles emerge, including preferential regulation of functionally important signaling nodes, integration of multiple regulatory mechanisms, and strong dependence on cellular and immunological context. Emerging evidence further suggests that some lncRNAs may act on defined protein states or regulatory interfaces without altering total protein abundance, although the generality of such functional selectivity remains to be established. Together, these findings support a hierarchical model in which lncRNAs translate specific molecular interactions into changes in protein function and, ultimately, innate immune states. This framework provides a basis for understanding how lncRNA–protein interactions contribute to the fine-tuning of innate immunity and for evaluating their potential as targets for selective immune modulation.

## 1 Introduction

Only a small fraction of the human genome encodes proteins, whereas extensive transcription occurs across non-protein-coding regions, giving rise to a diverse repertoire of non-coding RNAs (ncRNAs) (1). Among these, long non-coding RNAs (lncRNAs), generally defined as transcripts longer than 200 nucleotides with little or no protein-coding capacity, represent one of the most abundant and diverse classes of ncRNAs (2–4). Most lncRNAs are transcribed by RNA polymerase II and possess several mRNA-like features, including 5′ capping and 3′ polyadenylation. They can originate from diverse genomic regions, including intergenic regions, introns, antisense strands, and pseudogenes (5). Although lncRNAs generally exhibit relatively low sequence conservation, they often display marked tissue-, cell type-, and developmental stage-specific expression patterns, highlighting their important roles in fine-tuning gene regulatory networks (6, 7).

Functionally, lncRNAs can regulate cellular processes through interactions with DNA, RNA, or proteins, with direct interactions with proteins representing an important layer of lncRNA-mediated regulation (8, 9). In the nucleus, lncRNAs can interact with transcription factors, chromatin-remodeling complexes, and heterogeneous nuclear ribonucleoproteins to reshape chromatin states or influence the assembly of transcriptional complexes (10). In the cytoplasm, lncRNAs interact with RNA-binding proteins and key signaling effectors to regulate mRNA splicing, stability, and translation, thereby altering protein composition and downstream signal transduction (11, 12). These diverse regulatory modes enable lncRNAs to function as context-dependent regulators linking RNA metabolism to cellular signaling.

Innate immunity constitutes the first line of host defense against invading pathogens and relies on the coordinated activity of pattern recognition receptors (PRRs), downstream signaling pathways, and effector molecules or effector cells (13, 14). PRRs recognize pathogen-associated molecular patterns (PAMPs), such as lipopolysaccharide (LPS) and viral nucleic acids, as well as damage-associated molecular patterns (DAMPs), thereby initiating innate immune responses (15). Major PRR families include Toll-like receptors (TLRs), RIG-I-like receptors (RLRs), NOD-like receptors (NLRs), AIM2-like receptors (ALRs), and C-type lectin receptors (CLRs), which are distributed across the plasma membrane, endosomal compartments, or cytoplasm and recognize distinct pathogen-associated signals (16–18). Following PRR activation, pathway-specific adaptor proteins relay signals to downstream transcription factors. TLRs primarily signal through MyD88 or TRIF, RLRs through MAVS, and cytosolic DNA-sensing pathways through STING, with these pathways converging on NF-κB and/or IRF3/7 to drive the production of pro-inflammatory cytokines, chemokines, and type I interferons (IFN-I; IFN-α/β) (16, 17, 19–21). Secreted IFN-I subsequently binds to its cell-surface receptors and activates the Janus kinase (JAK)/signal transducer and activator of transcription (STAT) pathway, thereby inducing the expression of interferon-stimulated genes (ISGs) and establishing an antiviral state (22, 23). In parallel, NLRs can assemble inflammasomes, leading to activation of caspase-1 and cleavage of the precursors of interleukin-1β and interleukin-18 (IL-1β/IL-18), thereby reinforcing inflammatory responses against microbial infection (24).

Innate immune signaling is tightly controlled at multiple levels, including protein abundance, post-translational modifications (PTMs), subcellular localization, and protein–protein interactions (20, 21, 25). Increasing evidence has established lncRNAs as important regulators of innate immunity (26–30). The expanding catalog of lncRNAs identified through transcriptomic studies has provided a broad basis for investigating their functional roles (31), including in immune cell types and infection contexts (26–30, 32, 33). lncRNAs can influence immune responses through diverse mechanisms, including interactions with DNA, RNA, and proteins. Some act as competing endogenous RNAs (ceRNAs) by sequestering microRNAs (miRNAs), whereas others interact directly with regulatory proteins or modulate upstream factors that determine protein activity, stability, or localization (32, 34). Immune-regulatory lncRNAs can also exert context-dependent activating and repressive effects on response genes (35). Among these mechanisms, direct or protein-centered regulation is particularly relevant to innate immune signaling because it can alter the functional state of signaling proteins without necessarily requiring changes in their expression.

Despite the growing number of individual studies, previous reviews have surveyed lncRNA functions across innate immunity and host–virus interactions, often integrating DNA-, RNA-, and protein-level regulatory mechanisms (33). A review focused specifically on how lncRNA–protein interactions shape innate immune signaling therefore remains warranted. Here, we prioritize mechanistically resolved studies in which an lncRNA has been linked to a defined protein interaction or protein-level consequence and to an immune or antiviral phenotype. Because the evidence base remains heterogeneous, a small number of non-canonical immune examples are retained as explicitly identified comparators when they illuminate a transferable protein-regulatory principle; they are not treated as direct evidence for canonical innate immune signaling.

Mechanistic evidence for lncRNA–protein regulation varies substantially in depth. Association detected by RNA pull-down, RNA immunoprecipitation, or related enrichment assays does not by itself establish direct binding or functional causality. Stronger support comes from combining orthogonal interaction assays with binding-site or domain mapping, mutational analysis, endogenous perturbation, and rescue. CLIP-based and proximity-labeling approaches can further resolve RNA–protein interactions in their native cellular context and improve spatial or temporal resolution (36–38).

In this review, we adopt a protein-centered perspective to examine how lncRNA–protein interactions shape innate immune responses. Rather than cataloguing individual lncRNAs according to specific immune pathways, we organize the available evidence within a hierarchical framework that links molecular regulatory mechanisms to protein-level consequences and, ultimately, immune outcomes. At the mechanistic level, lncRNAs can regulate post-translational modifications, alter protein conformations, and remodel protein–protein interactions. These molecular events can subsequently influence protein stability, subcellular localization, molecular activity, and protein–protein interaction-dependent signal transmission, thereby altering the magnitude, duration, and spatial organization of innate immune responses. We further discuss common principles that emerge across these regulatory modes, including preferential targeting of functionally important signaling nodes, integration of multiple mechanisms, dependence on cellular and immunological context, and the emerging possibility of functional selectivity. By connecting molecular interactions with protein functional states and immune outcomes, this framework provides an integrated view of how lncRNAs fine-tune innate immune signaling.

## 2 Molecular mechanisms of lncRNA-mediated protein regulation in innate immunity

These regulatory dimensions are mechanistically interconnected and should not be interpreted as mutually exclusive biological categories. A single lncRNA–protein regulatory axis may influence several protein properties sequentially or simultaneously; for example, remodeling of a protein–protein interaction can alter a post-translational modification, which may subsequently affect protein activity, stability, localization, or downstream signal transmission. For analytical clarity, we therefore classify representative examples according to the predominant protein-level consequence emphasized by the available experimental evidence: protein stability, subcellular localization, molecular activity, or protein–protein interaction-dependent signal transmission. When an individual regulatory axis spans more than one dimension, the additional effects are explicitly acknowledged in the text and tables. Thus, the four categories are used as organizing axes within a hierarchical framework rather than as rigid or mutually exclusive mechanistic classes. This framework emphasizes how lncRNAs regulate innate immune signaling at the level of pre-existing proteins and their functional states, rather than solely through transcriptional or post-transcriptional control of gene expression.

### 2.1 lncRNA-mediated regulation of protein stability in innate immunity

Protein stability is an important determinant of the abundance and functional availability of signaling proteins and can thereby influence the strength and duration of innate immune responses. lncRNAs can affect innate immune signaling by altering the stability and turnover of specific proteins, with effects that may either enhance or restrain antiviral and antibacterial responses. These regulatory effects can arise through direct or indirect mechanisms. Some lncRNAs interact with target proteins or components of their regulatory machinery and alter post-translational modifications, including ubiquitination, thereby affecting protein degradation (39–42). Others act through upstream regulators of protein turnover and indirectly change the abundance of downstream signaling proteins. The representative regulatory axes discussed in this section are summarized in **Figure 1** and **Table 1**.

**Figure 1:**
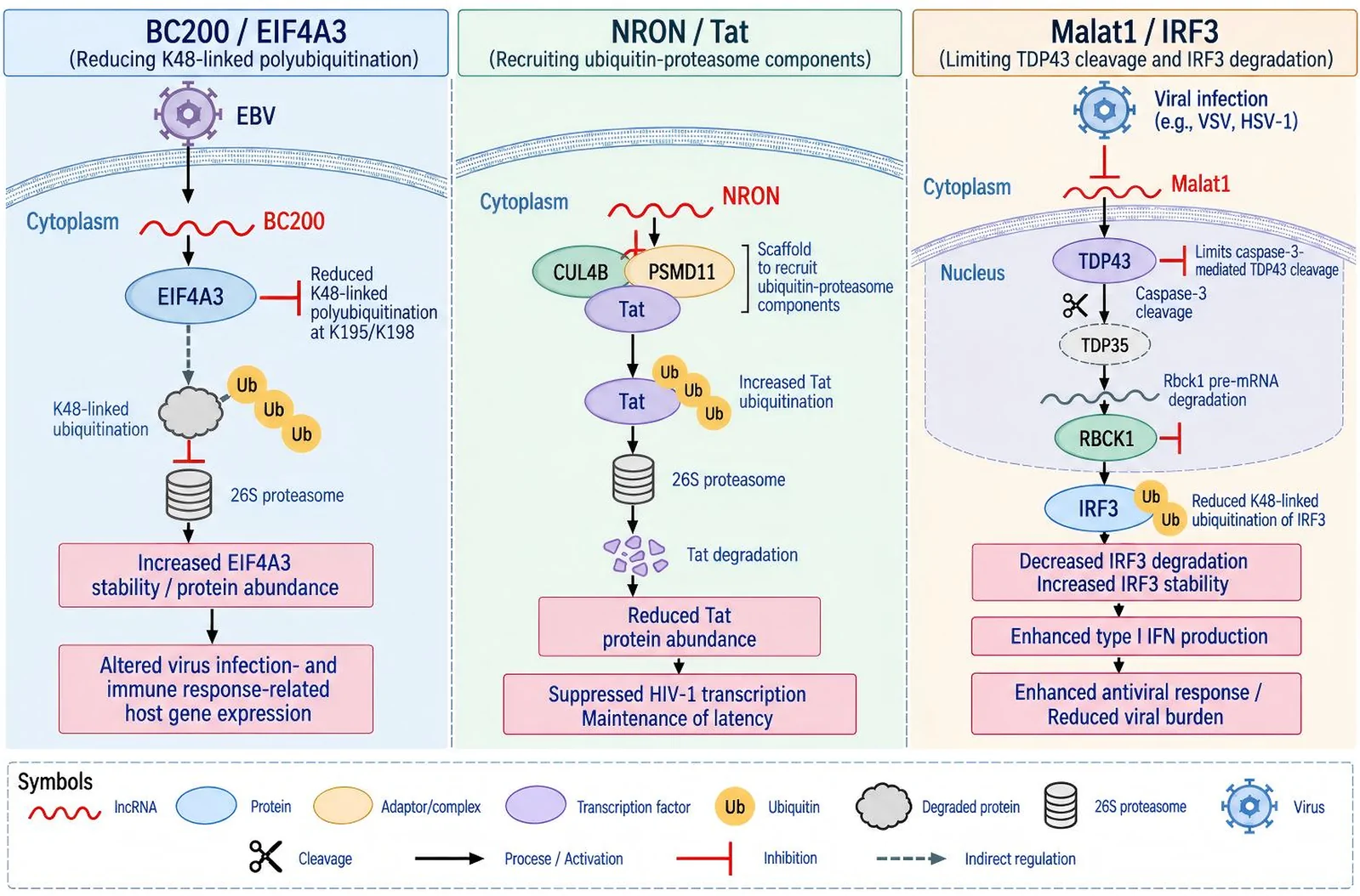
Direct and indirect regulation of protein stability by lncRNAs in innate immune responses. Schematic overview of representative mechanisms by which lncRNAs regulate protein ubiquitination, proteasomal degradation, and protein stability in distinct immune or viral contexts. Representative examples include BC200-mediated reduction of EIF4A3 K48-linked polyubiquitination, NRON-mediated recruitment of Tat to ubiquitin–proteasome components, and indirect regulation of IRF3 stability through the Malat1–TDP43–TDP35–RBCK1 cascade. Together, these examples show that lncRNAs can influence immune and antiviral responses by acting at distinct points in protein-stability control.

**Table 1:** Representative lncRNA-mediated regulation of protein stability in innate immunity and related immune contexts.

lncRNA	Target protein	Regulation method	Immune/antiviral consequence	Related pathogens/stimuli	Reference literature
Malat1	TDP43/TDP35/RBCK1/IRF3	Regulates IRF3 stability via the TDP43–TDP35–RBCK1 axis	Modulates IFN-I responses	VSV, HSV-1	(41)
BC200	EIF4A3	Reduces K48-linked polyubiquitination of EIF4A3 at K195/K198	Alters EBV-associated host gene-expression programs; immune-response pathways are enriched among affected genes	EBV	(39)
NRON	Tat	Bridges Tat to CUL4B/PSMD11-containing ubiquitin–proteasome components and facilitates Tat ubiquitination/degradation	Promotes Tat degradation and suppresses HIV-1 transcription	HIV	(51)
LINC2781	G3BP2	Prevents STAT1 degradation	Enhances antiviral immunity	CVB5	(42)
ZFAS1	ZNFX1	Maintains ZNFX1 protein stability; degradation machinery remains undefined	Promotes antiviral innate immunity	RNA/DNA viruses	(50)
USP30-AS1	PHB1	Stabilizes PHB1	Promotes IAV replication	IAV	(40)
VINR	Cactin	Prevents Cactin degradation	Promotes innate immune responses	Drosophila C virus/bacteria	(49)
NEAT1	STAT6	Interacts with STAT6 and inhibits its ubiquitination; ubiquitin linkage/site not defined in the cited study	Promotes Th2 differentiation	CD4+ T cells; Th2-associated immune context	(52)

#### 2.1.1 lncRNA-mediated regulation of ubiquitin-dependent protein stability

Ubiquitin-dependent protein turnover is a major determinant of the abundance and duration of innate immune signaling proteins (43–48). By interacting directly with target proteins or with components of the ubiquitin–proteasome system, lncRNAs can alter the balance between protein degradation and preservation, thereby influencing the magnitude of antiviral or inflammatory responses. Several studies have identified lncRNAs that regulate this process by interfering with ubiquitination or by limiting the access of E3 ubiquitin ligases to their protein substrates.

BC200 provides a direct example of lncRNA-mediated control of protein stability through ubiquitination. During Epstein–Barr virus (EBV) infection, BC200 directly binds EIF4A3 and reduces K48-linked polyubiquitination at K195 and K198, thereby limiting proteasomal turnover and increasing EIF4A3 protein stability (39). BC200 or EIF4A3 depletion alters the expression of host genes enriched in virus-infection and immune-response pathways, linking the BC200–EIF4A3 axis to infection-associated host gene regulation. However, a direct effect on a defined innate immune signaling pathway has not yet been established.

During influenza A virus (IAV) infection, USP30-AS1 is upregulated through JAK–STAT signaling and is exploited by the virus to suppress host antiviral responses. USP30-AS1 directly binds PHB1 and sequesters it from the E3 ubiquitin ligase TRIM21, thereby preventing ubiquitin-dependent PHB1 degradation and increasing PHB1 stability (40). Stabilized PHB1, together with USP30-AS1, promotes PHB1–IRF3 association, which impairs IRF3 nuclear translocation and ultimately facilitates IAV replication. Thus, USP30-AS1 couples protection of PHB1 from ubiquitin-dependent turnover to suppression of IRF3-mediated antiviral signaling.

A related mechanism operates in Drosophila during Drosophila C virus (DCV) infection. The DCV viral suppressor of RNA interference (VSR) promotes accumulation of VINR, which interacts with Cactin and prevents its ubiquitin–proteasome-dependent degradation (49). Stabilized Cactin activates a non-canonical innate immune pathway that promotes antimicrobial peptide gene expression and restricts DCV replication. VINR deficiency therefore increases susceptibility to DCV infection, demonstrating that lncRNA-mediated stabilization of an immune regulator can directly enhance antiviral defense in invertebrates.

Together, these examples illustrate three related routes by which lncRNAs preserve protein abundance: BC200 reduces K48-linked polyubiquitination of EIF4A3, USP30-AS1 prevents E3 ligase access to PHB1, and VINR protects Cactin from ubiquitin–proteasome-dependent degradation. Despite their distinct molecular implementations, each mechanism converts an infection-associated lncRNA–protein interaction into altered protein stability and downstream host responses.

#### 2.1.2 lncRNA-mediated regulation of protein stability through indirect mechanisms

lncRNAs can also regulate protein stability indirectly by acting on intermediate factors that control protein ubiquitination or degradation. Rather than directly modifying the final immune effector, these lncRNAs alter upstream regulatory interactions or degradation machinery, thereby changing the abundance of downstream signaling proteins.

Malat1 provides a well-defined example. Malat1 is abundant under resting conditions but is significantly downregulated following viral infection, including VSV, EMCV, and HSV-1 infection. At baseline, nuclear Malat1 binds TDP43 and prevents its caspase-3-mediated cleavage into TDP35. Viral infection-induced Malat1 downregulation releases this inhibition, allowing TDP35 to accumulate and promote degradation of Rbck1 pre-mRNA. The resulting reduction in the E3 ubiquitin ligase RBCK1 decreases K48-linked ubiquitination and proteasomal degradation of IRF3, increasing nuclear IRF3 abundance and type I IFN production (41). Thus, Malat1 downregulation → TDP35 accumulation → RBCK1 reduction → IRF3 stabilization → enhanced IFN-I-mediated antiviral immunity.

During coxsackievirus B5 (CVB5) infection, LINC2781 is significantly upregulated and acts as a positive regulator of antiviral immunity. LINC2781 directly binds G3BP2 and prevents G3BP2-mediated ubiquitination and degradation of STAT1, thereby increasing STAT1 stability and sustaining JAK–STAT signaling (42). This promotes interferon-stimulated gene expression and suppresses CVB5 replication. Thus, CVB5-induced LINC2781 → inhibition of G3BP2-mediated STAT1 degradation → STAT1 stabilization → enhanced JAK–STAT/ISG responses → reduced viral replication.

ZFAS1 provides another positive-feedback mechanism. ZFAS1 is upregulated by both RNA and DNA viruses and by type I IFNs through JAK–STAT signaling. ZFAS1 associates with the antiviral dsRNA sensor ZNFX1 and increases its protein stability, although the precise degradation machinery involved has not yet been defined (50). Increased ZNFX1 stability enhances IRF3 activation, IFN-β and ISG expression, and antiviral activity. Accordingly, virus/IFN-I-induced ZFAS1 → increased ZNFX1 stability → enhanced antiviral signaling → increased IFN-I/ISG responses → restriction of viral infection.

Collectively, these mechanisms show that lncRNAs can regulate innate immunity by controlling the turnover of key signaling proteins either directly or through intermediate regulators. Importantly, the direction of the response is context dependent: virus-induced LINC2781 and ZFAS1 stabilize antiviral proteins and strengthen host defense, whereas virus-induced USP30-AS1 stabilizes PHB1 to suppress IRF3 signaling and facilitate IAV replication. Conversely, viral downregulation of Malat1 relieves an inhibitory pathway, stabilizing IRF3 and enhancing type I IFN production.

### 2.2 lncRNA-mediated regulation of protein localization in innate immunity

The subcellular localization of signaling proteins is closely linked to their function because compartmentalization determines access to signaling partners, substrates, and transcriptional targets. Nucleocytoplasmic transport through nuclear pore complexes is a tightly regulated process that controls the movement of proteins between the cytoplasm and nucleus (53–55). Beyond changes in protein abundance, alterations in protein localization can therefore provide an additional means of controlling the timing, magnitude, and duration of intracellular signaling. lncRNAs can regulate this spatial dimension of innate immunity by influencing nucleocytoplasmic transport, intracellular retention, or recruitment of signaling proteins to specific cellular compartments. These mechanisms can determine where a signaling protein is available to interact with its upstream regulators or downstream effectors, thereby reshaping immune signaling without necessarily changing its overall abundance (56) (**Figure 2**). Representative examples are summarized in **Table 2**.

**Figure 2:**
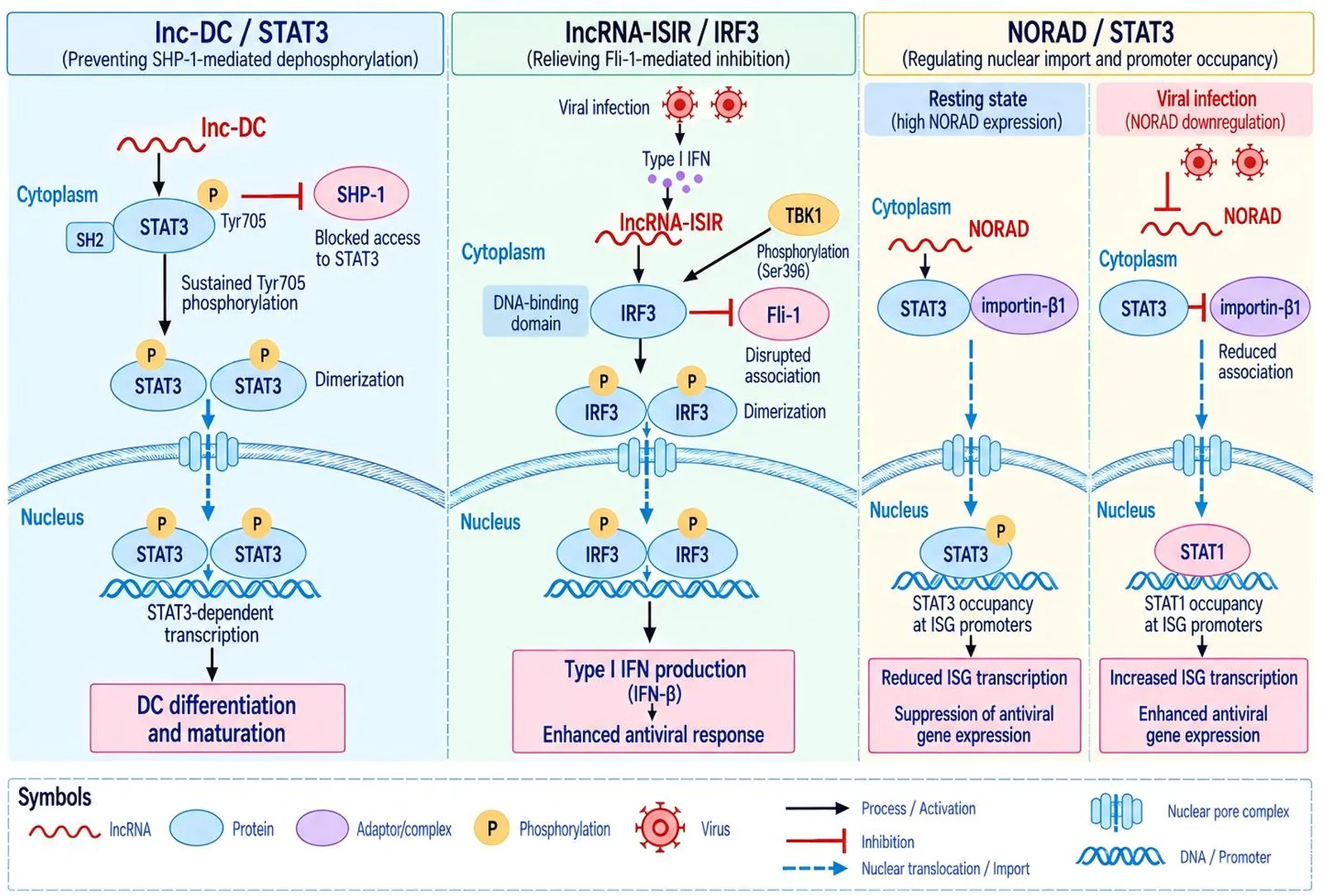
Regulation of protein subcellular localization by lncRNAs in innate immune responses. Schematic overview of representative mechanisms by which lncRNAs regulate the subcellular localization of immune-related proteins through phosphorylation-dependent activation or modulation of nuclear transport machinery. Representative examples include lnc-DC-mediated maintenance of STAT3 Tyr705 phosphorylation with downstream nuclear accumulation, lncRNA-ISIR mediated promotion of IRF3 activation and nuclear translocation, and NORAD-dependent enhancement of the STAT3–importin-β1 association and STAT3 nuclear localization. These mechanisms illustrate how lncRNAs can influence immune and antiviral responses by altering the spatial availability of signaling proteins.

**Table 2:** Representative lncRNA–protein regulatory axes controlling protein localization in innate immunity and related immune contexts.

lncRNA	Target protein	Regulation method	Immune/antiviral consequence	Related pathogens/stimuli	Reference literature
lnc-DC	STAT3	Binds STAT3 and prevents SHP-1-mediated Tyr705 dephosphorylation; nuclear accumulation is downstream of sustained STAT3 activation	Promotes dendritic-cell differentiation	Dendritic-cell differentiation	(60)
lncRNA-ISIR	IRF3/Fli-1	Binds the IRF3 DNA-binding domain, removes inhibitory Fli-1 association, and facilitates IRF3 phosphorylation, dimerization, and nuclear translocation	Enhances type I IFN production and antiviral responses	viral infection, IFN-I stimulation	(61)
NORAD	STAT3	Promotes STAT3–importin-β1 association and STAT3 nuclear localization; alters STAT3/STAT1 occupancy at ISG promoters	Inhibits antiviral immunity	viral infection	(63)
NRON	NFAT	Anchors NFAT in the cytoplasm	Inhibits T cell activation	T cell activation signal	(67)
NEAT1	SFPQ	Promotes relocation of SFPQ from the IL8 promoter to NEAT1-containing paraspeckles	Relieves SFPQ-mediated IL8 repression in the studied immune-stimulus context	IAV, HSV-1, poly(I:C)	(64)
lncRNA-BTX	DHX9/ILF3/	Strengthens DHX9–JMJD6 and ILF3–ILF2 interactions, favoring DHX9 cytoplasmic redistribution and ILF3 nuclear retention	Inhibits host antiviral defense	viral infection	(66)
lncBST2-2	IRF3	Promotes IRF3 nuclear translocation	Promotes the production of type I IFN	HCV, NDV, VSV, HSV; IFN/PRR stimulation	(68)

#### 2.2.1 lncRNA-mediated regulation of nuclear import through phosphorylation

Nuclear localization is frequently controlled by reversible phosphorylation, which can alter the interaction of signaling proteins with nuclear transport machinery and thereby influence their nuclear availability (57, 58). Nuclear import generally depends on karyopherin family transport receptors that recognize cargo proteins directly or through adaptor proteins and mediate their passage through nuclear pore complexes (55, 59). lncRNAs can exploit this regulatory layer by interacting with signaling proteins and altering their phosphorylation state or accessibility to regulatory enzymes, thereby promoting or restricting subsequent nuclear translocation.

The dendritic cell-specific lncRNA lnc-DC provides an example of phosphorylation-dependent control of protein localization. lnc-DC binds STAT3 in the cytoplasm and prevents STAT3 association with, and dephosphorylation by, SHP-1, thereby maintaining phosphorylation at Tyr705 (60). Sustained STAT3 activation is accompanied by increased nuclear accumulation and transcriptional activity during dendritic-cell differentiation. Thus, lnc-DC is best viewed as an indirect regulator of STAT3 localization: the directly demonstrated molecular event is protection of STAT3 Tyr705 phosphorylation, whereas nuclear accumulation occurs downstream of sustained STAT3 activation.

A related mechanism has been described for the interferon-inducible lncRNA-ISIR directly binds the DNA-binding domain of IRF3 in the cytoplasm and facilitates IRF3 activation by promoting its phosphorylation and dimerization, thereby favoring subsequent nuclear translocation (61, 62). Through this interaction, lncRNA-ISIR enhances type I interferon production and antiviral innate immune responses (61). Both cases show that lncRNA–protein interactions can regulate protein localization by altering the phosphorylation-dependent transition between cytoplasmic and nuclear signaling states (60, 61).

#### 2.2.2 Direct regulation of protein localization through non-modification mechanisms

lncRNAs can also regulate protein localization without directly altering the phosphorylation or other post-translational modification states of the target protein. Instead, they can modulate the interaction between signaling proteins and nuclear transport factors or reorganize intracellular compartments that retain or redistribute regulatory proteins. Such mechanisms allow lncRNAs to control the spatial availability of signaling proteins independently of changes in their overall abundance.

NORAD provides an example of lncRNA-mediated regulation of nuclear import through the cellular transport machinery. NORAD interacts with STAT3, and NORAD depletion reduces the association between STAT3 and importin-β1, consistent with a role for NORAD in promoting STAT3 nuclear localization (63). In cells with higher NORAD, nuclear STAT3 occupies selected interferon-stimulated gene promoters and is associated with reduced transcription; NORAD depletion decreases STAT3 promoter occupancy while increasing STAT1 occupancy and antiviral transcription. Following viral infection, NORAD expression is reduced, which is accompanied by lower nuclear STAT3 and stronger STAT1-dependent antiviral responses. These data support regulation of STAT3 nuclear availability and promoter occupancy rather than a direct inhibitory interaction between STAT3 and STAT1.

NEAT1 provides a distinct form of spatial regulation by organizing a specialized nuclear compartment. NEAT1 is an essential structural component of nuclear paraspeckles and interacts with paraspeckle-associated proteins such as SFPQ. Upon viral infection or poly(I:C) stimulation, NEAT1 expression and paraspeckle formation increase, promoting the relocation of SFPQ from the IL8 promoter to paraspeckles (64). Because SFPQ functions as a transcriptional repressor at the IL8 promoter, its redistribution reduces this repression and facilitates IL8 transcription. Thus, NEAT1 does not simply alter the localization of an individual protein; it reorganizes a nuclear microenvironment in which the functional availability of SFPQ is changed. This example highlights how lncRNA-dependent compartmentalization can convert changes in protein localization into altered innate immune gene expression. This mechanism places the NEAT1–SFPQ axis within a broader role for virus-responsive paraspeckles in organizing nuclear immune regulation (65).

#### 2.2.3 Coordinated regulation of bidirectional nucleocytoplasmic protein transport

Some lncRNAs can coordinate the localization of multiple regulatory proteins, thereby producing a broader spatial effect on innate immune signaling than regulation of a single transport event. Virus-induced lncRNA-BTX provides an example of such coordinated spatial regulation. Following viral infection, lncRNA-BTX enhances the interaction between DHX9 and JMJD6, promoting the redistribution of DHX9 from the nucleus to the cytoplasm and reducing its contribution to antiviral gene regulation. At the same time, lncRNA-BTX enhances the interaction between ILF3 and ILF2, promoting nuclear retention of ILF3 and limiting its cytoplasmic functions in viral RNA metabolism (66).

These observations indicate that lncRNAs can coordinate the intracellular distribution of multiple regulatory proteins rather than controlling a single nuclear import or export event. By simultaneously influencing nuclear export and nuclear retention, lncRNA-BTX can reshape the spatial organization of antiviral signaling. This type of multi-protein regulation expands the functional range of lncRNA-mediated spatial control and illustrates how an lncRNA can coordinate multiple protein interactions and localization events within the same signaling network.

### 2.3 lncRNA-mediated regulation of protein activity in innate immunity

The activity of innate immune signaling proteins is determined not only by their abundance or subcellular localization but also by their conformational state, post-translational modifications, and interactions with regulatory partners. lncRNAs can influence these functional states through direct binding to signaling proteins or by modulating the enzymes and complexes that control their activation. Such regulation can either restrain signaling, as in the maintenance of inactive PRR conformations, or enhance signaling by promoting activating modifications and protein interactions (**Figure 3**). Representative examples of these mechanisms are summarized in **Table 3**.

**Figure 3:**
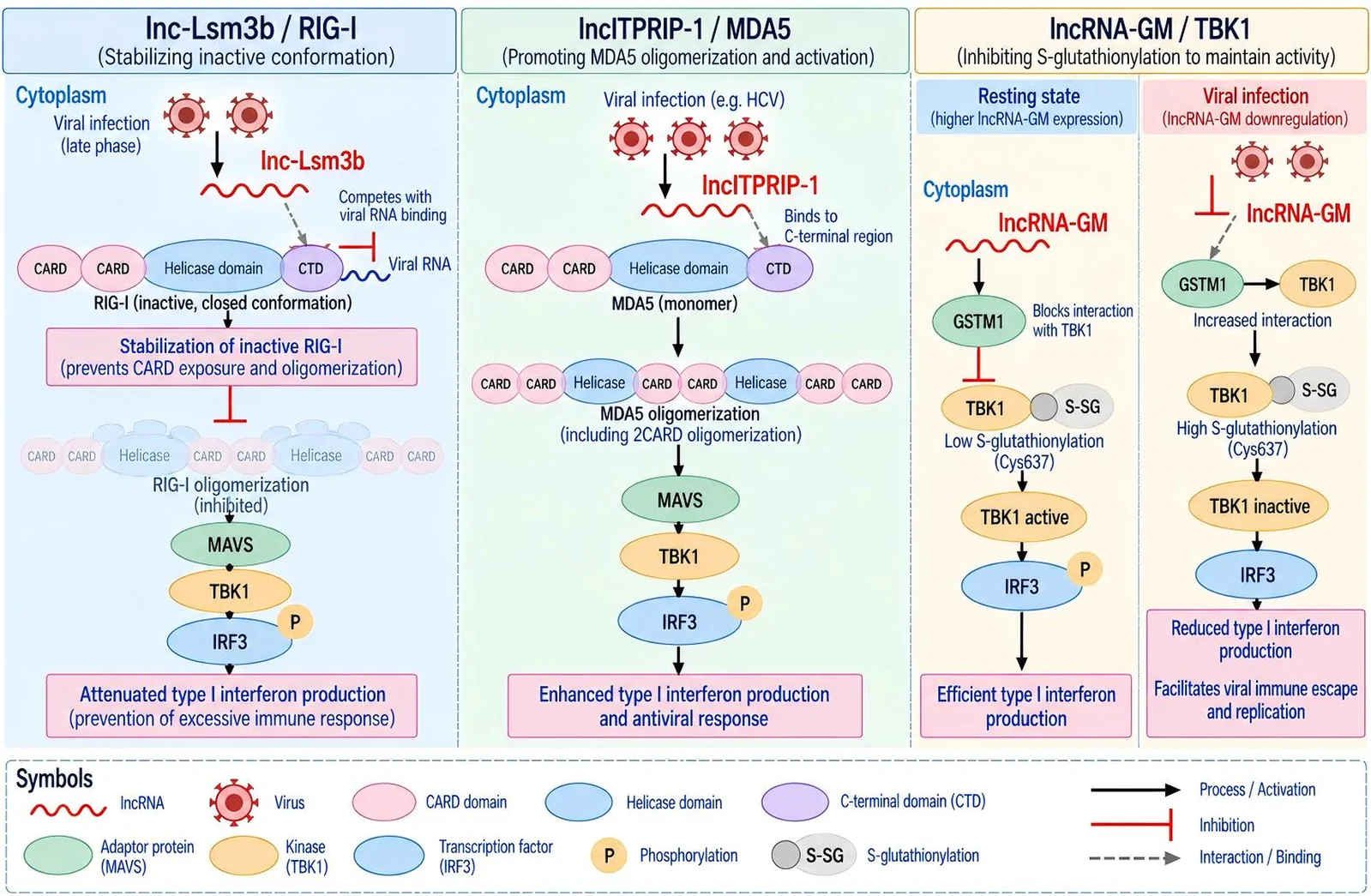
Regulation of protein activity by lncRNAs in innate immunity. Schematic overview of representative mechanisms by which lncRNAs regulate the activity of innate immune signaling proteins through conformational or oligomerization control and modulation of post-translational modifications. Representative examples include lnc-Lsm3b-mediated stabilization of an inactive RIG-I state, lncITPRIP-1-mediated promotion of MDA5 oligomerization, and lncRNA-GM-mediated preservation of TBK1 activity by reducing inhibitory S-glutathionylation. These mechanisms illustrate how lncRNA–protein interactions can restrain or enhance antiviral signaling by altering defined activation-associated protein states.

**Table 3:** Summary of lncRNA-mediated regulation of protein activity in innate immunity.

lncRNA	Target protein	Regulation method	Immune/antiviral consequence	Related pathogens/stimuli	Reference literature
lnc-Lsm3b	RIG-I	Competes with viral RNA for the RIG-I CTD and stabilizes an autorepressed monomeric state	Suppresses IFN-I	VSV, SeV; RNA-virus response (VSV validated *in vivo*)	(73)
lncITPRIP-1	MDA5	Binds the MDA5 C terminus and promotes MDA5/2CARD oligomerization and activation; reported as K63-polyubiquitination-independent	Promotes IFN-I	HCV, VSV, SeV, HSV-1	(74)
Lnczc3h7a	RIG-I	Binds TRIM25 and activated RIG-I, stabilizes their complex, and facilitates TRIM25-mediated K63-linked RIG-I ubiquitination	Promotes IFN-I and antiviral signaling	VSV, SeV; VSV/IAV *in vivo*	(82)
lncNSPL	RIG-I	Competes with TRIM25 for binding to RIG-I and inhibits TRIM25-mediated K63-linked ubiquitination of RIG-I	Suppresses IFN-I	IAV (H1N1/H3N2)	(86)
lncRNA-GM	GSTM1/TBK1	Binds GSTM1, blocks GSTM1–TBK1 interaction, and reduces inhibitory TBK1 S-glutathionylation at Cys637	Promotes IFN-I	VSV, SeV, HSV-1, vaccinia virus	(87)
lncLrrc55-AS	PME-1/PP2A/IRF3	Promotes PME-1–PP2A interaction, leading to PP2A inactivation and sustained IRF3 phosphorylation	Promotes IFN-I	VSV, SeV, HSV-1, IAV	(88)
NKILA	IκBα/NF-κB	Directly binds IκBα and masks its IKK phosphorylation sites, preventing IκBα degradation	Suppresses NF-κB	Inflammatory/NF-κB stimulation	(90)
Lethe	RelA/p65	Directly interacts with RelA and inhibits its binding to NF-κB target DNA	Suppresses NF-κB-dependent inflammatory signaling	TNF-α, IL-1β, dexamethasone	(91)

#### 2.3.1 Conformational and oligomerization control of pattern-recognition receptors

Pattern-recognition receptors provide particularly clear examples of lncRNA-mediated control of protein conformation. RIG-I activation requires a transition from an autoinhibited conformation to an activated state that permits CARD exposure, oligomerization, and subsequent MAVS signaling (69–72). lnc-Lsm3b acts as an inducible negative regulator of this process. It binds the C-terminal domain of RIG-I, competes with viral RNA for RIG-I binding, and stabilizes an inactive conformation, thereby preventing inappropriate RIG-I activation and limiting excessive type I interferon production during the later phase of antiviral responses (73).

In contrast, lncITPRIP-1 positively regulates MDA5 activation. This lncRNA binds the C-terminal region of MDA5 and promotes MDA5 oligomerization, including oligomerization of the 2CARD region, in a manner reported to be independent of K63-linked polyubiquitination (74). These changes enhance MDA5-dependent interferon signaling. The importance of oligomerization as a regulatory checkpoint is independently supported by TRIM65-mediated K63-linked ubiquitination of MDA5, which promotes MDA5 oligomerization and activation (75). The available data therefore support promotion of MDA5 oligomerization and activation, rather than a defined lncITPRIP-1-induced conformational transition analogous to that established for RIG-I.

This mode of regulation is particularly relevant to RIG-I-like receptor signaling because activation depends on conformational transitions rather than on protein abundance alone. Structural studies of RIG-I have established that RNA recognition, CARD exposure, and oligomerization are tightly coupled steps in receptor activation (76–79). lncRNAs therefore have the potential to regulate innate immune signaling at an early molecular checkpoint by selectively stabilizing receptor states.

#### 2.3.2 Post-translational regulation of signaling protein activity

lncRNAs can also control protein activity by regulating post-translational modifications, which form a major layer of signal integration in cellular signaling (80). Phosphorylation is a particularly important example of such regulatory control (81). Lnczc3h7a illustrates how protein–protein interaction remodeling can be coupled to post-translational regulation of protein activity. Following viral infection, Lnczc3h7a simultaneously interacts with TRIM25 and activated RIG-I, thereby facilitating the formation of the TRIM25–RIG-I complex. This spatial organization promotes TRIM25-mediated K63-linked ubiquitination of RIG-I and enhances downstream RIG-I signaling through MAVS, TBK1, and IRF3 (82). Here, the lncRNA does not simply increase the abundance of RIG-I or TRIM25; instead, it promotes an activating modification by organizing the relevant enzymatic machinery around its substrate. RIG-I ubiquitination is regulated by multiple E3 ligases, and the relative contribution of TRIM25 and other ligases such as Riplet varies across experimental and cellular contexts (83). TRIM25 is itself an RNA-binding E3 ubiquitin ligase, and recent biochemical and iCLIP-based work shows that RNA binding contributes to its antiviral activity (84, 85). Accordingly, the Lnczc3h7a axis spans both protein–protein interaction-dependent complex assembly and post-translational regulation; it is discussed here under molecular activity because its predominant functional consequence is enhanced ubiquitination-dependent activation of RIG-I.

The opposite regulatory strategy is observed with lncNSPL. lncNSPL directly interacts with RIG-I and interferes with the association between RIG-I and TRIM25. As a consequence, K63-linked ubiquitination of RIG-I is reduced, resulting in attenuation of RIG-I activation and impaired antiviral signaling (86). The opposing effects of Lnczc3h7a and lncNSPL on the same signaling node provide an instructive example of how lncRNAs can establish either positive or negative control over an identical molecular checkpoint.

lncRNA-GM provides another example in which remodeling of a protein–protein interaction is coupled to altered post-translational regulation of protein activity. lncRNA-GM binds GSTM1 and interferes with the GSTM1–TBK1 interaction, thereby reducing GSTM1-mediated S-glutathionylation of TBK1 at Cys637. Because this modification negatively affects TBK1 kinase activity, limiting it preserves TBK1 signaling and promotes IRF3 phosphorylation and type I interferon production (87). This example expands the range of protein modifications regulated by lncRNAs beyond ubiquitination and phosphorylation. This axis therefore intersects the protein–protein interaction and molecular-activity dimensions but is discussed here according to its predominant consequence for TBK1 catalytic activity.

lncLrrc55-AS provides another example of indirect control of phosphorylation-dependent signaling. This interferon-inducible cytoplasmic lncRNA interacts with protein phosphatase methylesterase 1 (PME-1) and promotes its association with PP2A. The resulting alteration of PP2A methylation and holoenzyme assembly reduces PP2A phosphatase activity, thereby limiting dephosphorylation of IRF3 and sustaining IRF3 phosphorylation (88). Thus, the lncRNA regulates the phosphorylation state of IRF3 indirectly by controlling the activity of a phosphatase complex.

Taken together, these studies demonstrate that lncRNAs can regulate immune protein activity by controlling enzymes, substrates, and molecular complexes involved in post-translational modification. In several studied systems, the reported effect is modification- or interaction-specific; however, this should not be generalized to global functional selectivity unless alternative functions of the target protein have been directly tested.

#### 2.3.3 Remodeling of protein interaction interfaces

lncRNAs can further regulate protein activity by modifying the interaction interfaces through which signaling proteins communicate with their regulatory partners. LINC1392, for example, directly interacts with the RNA-binding protein ELAVL1 (HuR) and disrupts the ELAVL1–MDA5 interaction. This relieves ELAVL1-mediated inhibition of MDA5 and enhances antiviral signaling following viral infection (89). In this case, the functional consequence arises primarily from remodeling a protein–protein interaction rather than from changing MDA5 abundance.

NKILA represents another form of interface-dependent regulation. NKILA directly binds IκBα and interferes with the phosphorylation of Ser32 and Ser36 required for IκBα degradation. As a result, IκBα remains associated with NF-κB and restricts NF-κB nuclear translocation, thereby limiting inflammatory signaling (90). Although this mechanism involves regulation of phosphorylation and protein stability, the initial action of the lncRNA is direct recognition of a defined region of IκBα.

Lethe provides a distinct example involving transcription-factor activity. Lethe directly interacts with the RelA subunit of NF-κB and reduces its ability to bind target DNA. Induced by inflammatory signaling, Lethe therefore functions as a negative-feedback regulator that restrains excessive NF-κB-dependent transcription (91). Unlike mechanisms that alter protein abundance, Lethe directly modifies the transcriptional competence of an already existing signaling protein.

Additional antiviral examples broaden this interface-centered view. lncRNA#32 directly binds ATF2, and ATF2 is required for lncRNA#32-dependent interferon-stimulated gene expression and antiviral activity; hnRNPU also associates with and stabilizes lncRNA#32 (92). In human macrophages, CHROMR sequesters the transcriptional corepressor IRF2BP2, thereby supporting IRF-dependent interferon-stimulated gene expression during influenza A virus and SARS-CoV-2 infection (93). These examples illustrate protein-centered regulation at transcriptional interfaces without implying changes in target-protein abundance.

Collectively, these examples indicate that lncRNAs can regulate protein activity through at least three interconnected strategies: stabilization of specific conformational states, modulation of post-translational modifications, and remodeling of interaction interfaces. These mechanisms allow lncRNAs to control signaling output at the level of protein function rather than merely protein expression.

### 2.4 lncRNA-mediated regulation of protein–protein interactions and signaling transmission in innate immunity

Protein–protein interactions are central to innate immune signal transduction, where scaffold and adaptor proteins help organize pathway components (94). Following pathogen recognition, signaling information is transmitted through dynamic interactions among receptors, adaptor proteins, kinases, ubiquitin regulators, and transcription factors. The strength and organization of these interactions determine how efficiently signals are transmitted through the pathway. RNA-mediated control of protein complexes can involve scaffolding, recruitment, decoy activity, or spatial colocalization, and these mechanisms should not be treated as interchangeable (8, 95). lncRNAs can regulate this layer of signaling by promoting or disrupting specific protein interactions, serving as molecular scaffolds, or becoming structural components of larger signaling complexes. In this way, lncRNAs can alter the connectivity of signaling networks without necessarily changing the abundance of the individual proteins involved (**Figure 4**). Representative examples are summarized in **Table 4**.

**Figure 4:**
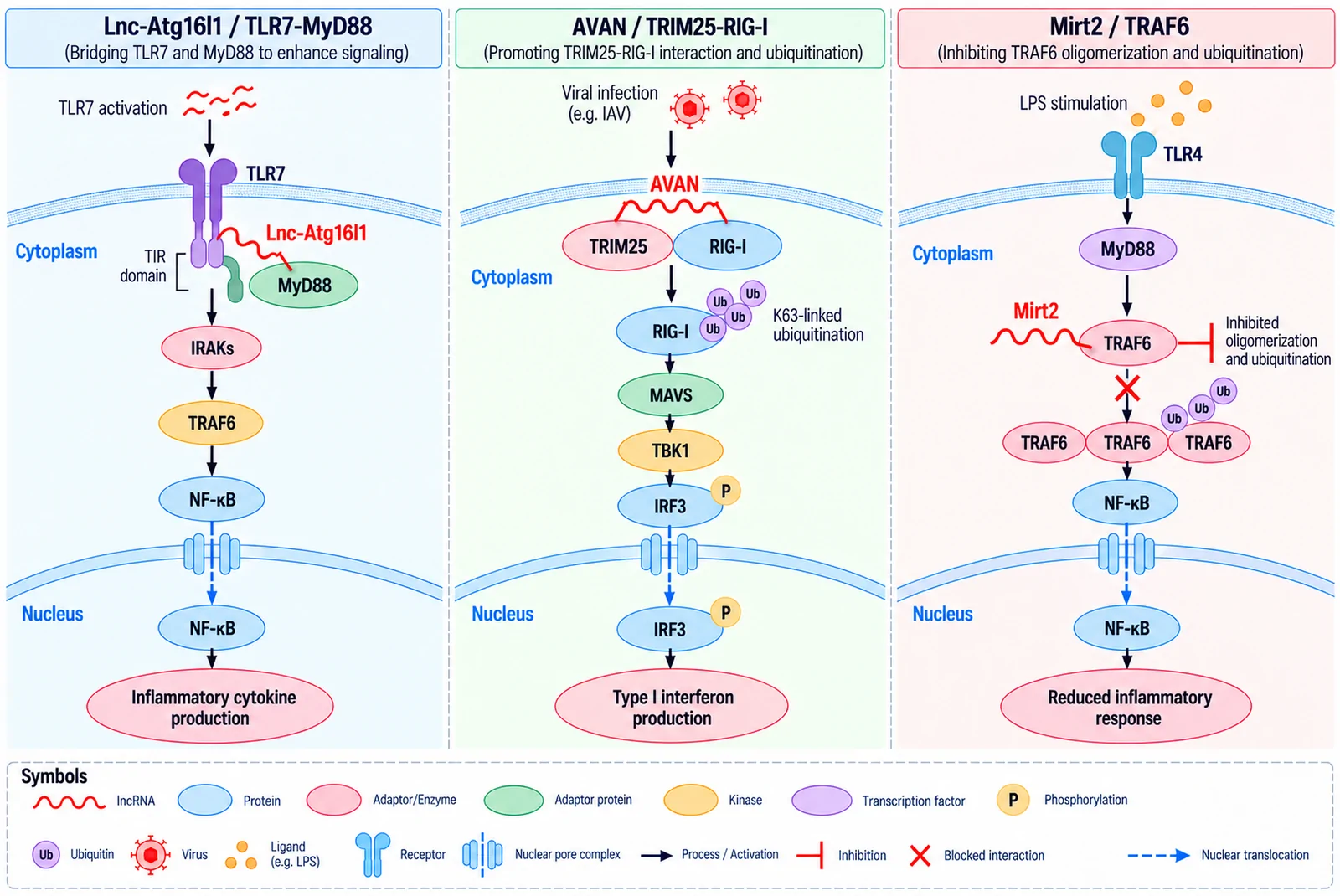
lncRNA-mediated regulation of protein–protein interaction-dependent signaling transmission in innate immunity. Schematic overview of representative mechanisms by which lncRNAs regulate innate immune signaling through modulation of protein–protein interactions and signaling complex assembly. Representative examples include Lnc-Atg16l1-mediated enhancement of the TLR7–MyD88 interaction, AVAN-mediated promotion of the TRIM25–RIG-I interaction and RIG-I K63-linked ubiquitination, and Mirt2-mediated inhibition of TRAF6 oligomerization and K63-linked autoubiquitination. These mechanisms illustrate how lncRNAs can function as molecular facilitators or inhibitory regulators of protein interactions to fine-tune inflammatory and antiviral signaling responses.

**Table 4:** Summary of lncRNA-mediated regulation of protein–protein interactions and signaling transmission in innate immunity.

lncRNA	Target protein	Regulation method	Immune/antiviral consequence	Related pathogens/stimuli	Reference literature
AVAN	TRIM25/RIG-I	Directly binds TRIM25 and enhances TRIM25–RIG-I association and K63-linked RIG-I ubiquitination; direct AVAN–RIG-I binding was not detected	Promotes IFN-I and ISG responses	IAV	(99)
Lnc-Atg16l1	TLR7/MyD88	Binds TLR7 and MyD88 at distinct RNA regions near U84 and A129, supporting TLR7–MyD88 complex assembly after TLR7 activation	Promotes TLR7-MyD88 inflammatory signaling	TLR7 activation; SLE model	(97)
RDUR	ILF2/ILF3; NF-κB	Interacts with ILF2/ILF3 and supports antiviral gene expression; NF-κB negative feedback is a parallel reported function	Promotes antiviral responses while limiting excessive inflammation	IAV, SeV, HSV-1, PRV	(102)
lncATV	RIG-I	Binds RIG-I, inhibits RIG-I-mediated signaling	Suppresses IFN-I, promotes viral replication	VSV, HCV, ZIKV, NDV, SeV	(100)
THRIL	hnRNPL	Forms an hnRNPL-containing RNP complex at the TNF-α promoter (boundary example; primarily transcriptional)	Promotes TNF-α expression and inflammatory response	Pam3CSK4 (TLR2/1 stimulation)	(103)
MaIL1	OPTN	Binds and stabilizes OPTN and functions as an RNA component of the TLR4–TRIF signaling machinery	Promotes TBK1-IRF3 signaling and type I IFN production	LPS (TLR4 stimulation)	(105)
LINC1392	ELAVL1	Binds ELAVL1 and blocks ELAVL1–MDA5 association, relieving ELAVL1-mediated negative regulation of MDA5	Activates MDA5-mediated IFN-I signaling, inhibits CVB5 replication	CVB5; viral RNA; IFN-β	(89)
Mirt2	TRAF6	Directly binds TRAF6 and inhibits its oligomerization and K63-linked auto-ubiquitination, thereby disrupting TRAF6-mediated signaling complex formation	Suppresses TLR4/MyD88-dependent NF-κB and MAPK activation, reduces pro-inflammatory cytokine production, and prevents excessive inflammatory responses	LPS	(101)
Neat1	NLRP3/NLRC4/AIM2 inflammasomes; caspase-1	Associates with NLRP3, NLRC4, and AIM2 inflammasomes, promotes inflammasome assembly and pro-caspase-1 processing, and stabilizes mature caspase-1	Promotes IL-1β production and pyroptosis	Inflammasome-activating stimuli; hypoxia	(98)
NEAT1	HEXIM1/DNA-PK/cGAS/PQBP1/STING	Binds HEXIM1 and is required for HDP-RNP assembly; the complex interacts with cGAS/PQBP1 and is remodeled by foreign DNA, leading to STING recruitment and DNAPKc/IRF3 activation	Promotes DNA-mediated innate immune signaling through the cGAS–STING–IRF3 pathway	Foreign DNA	(104)

#### 2.4.1 lncRNAs promoting signaling complex assembly

A major mechanism by which lncRNAs regulate protein–protein signaling is to facilitate the spatial organization and assembly of signaling complexes (96). Following TLR7 activation, Lnc-Atg16l1 undergoes stem-loop changes and binds TLR7 and MyD88 at distinct RNA regions near U84 and A129, respectively. These interactions increase after TLR7 activation and are required for full TLR7-dependent inflammatory signaling, supporting a bridging role for Lnc-Atg16l1 in the TLR7–MyD88 complex (97). A related mode of complex assembly operates in inflammasome signaling. In mouse macrophages, Neat1 associates with the NLRP3, NLRC4, and AIM2 inflammasomes and enhances their assembly and subsequent pro-caspase-1 processing. Upon stimulation with inflammasome-activating signals, Neat1 dissociates from nuclear paraspeckles and translocates to the cytoplasm, where it contributes to inflammasome activation. Neat1 also stabilizes mature caspase-1, thereby promoting IL-1β production and pyroptosis. These findings extend lncRNA-mediated regulation of signaling-complex assembly to NLR- and AIM2-associated inflammasomes (98). Similarly, the antiviral lncRNA AVAN is induced following influenza A virus infection and directly binds the E3 ubiquitin ligase TRIM25. AVAN enhances the TRIM25–RIG-I association and TRIM25-mediated K63-linked ubiquitination of RIG-I, thereby promoting downstream antiviral signaling (99). Importantly, the original study did not detect a direct AVAN–RIG-I interaction; AVAN should therefore not be depicted as a scaffold that directly binds both TRIM25 and RIG-I.

#### 2.4.2 lncRNAs as inhibitory regulators of protein–protein signaling

In contrast to scaffold-mediated signal amplification, some lncRNAs suppress innate immune signaling by interfering with productive protein interactions. Human-specific lncATV is a representative example. lncATV is predominantly cytoplasmic and associates with RIG-I. lncATV overexpression suppresses RIG-I-mediated antiviral signaling, whereas lncATV depletion enhances RIG-I signaling and restricts replication of several RNA viruses (100). These findings suggest that lncATV acts at the level of the RIG-I signaling complex to limit signal propagation rather than by substantially changing RIG-I abundance. Mirt2 represents another example of inhibitory regulation at the level of protein interactions. Following lipopolysaccharide (LPS) stimulation, Mirt2 is induced and directly binds TRAF6. This interaction inhibits TRAF6 oligomerization and K63-linked autoubiquitination, thereby interfering with the formation and activation of TRAF6-dependent signaling complexes. Consequently, downstream NF-κB and mitogen-activated protein kinase (MAPK) signaling is attenuated, leading to reduced production of pro-inflammatory cytokines and protection against excessive inflammatory responses (101).

#### 2.4.3 lncRNAs as structural components of signaling complexes

Some lncRNAs act not simply as positive or negative regulators of individual protein interactions but as structural RNA components that contribute to the organization and functional regulation of larger signaling complexes. RDUR interacts with the RNA-binding proteins ILF2 and ILF3 and contributes to the efficient expression of antiviral genes, including IFITM3 and MX1. In parallel, RDUR provides negative feedback to virus-induced NF-κB activation, thereby limiting excessive inflammatory responses. Thus, RDUR coordinates antiviral defense with inflammatory restraint during viral infection (102). THRIL forms a ribonucleoprotein complex with the RNA-binding protein heterogeneous nuclear ribonucleoprotein L (hnRNPL). This complex associates with the promoter region of the TNF-α gene and promotes TNF-α transcription, thereby contributing to inflammatory gene expression (103). THRIL primarily acts at the transcriptional level rather than by remodeling a canonical protein–protein signaling complex; it is therefore included here only as a boundary example of an lncRNA-organized ribonucleoprotein complex, not as core evidence for protein–protein signal transmission.A further example of lncRNA-dependent organization of an innate immune ribonucleoprotein complex is provided by NEAT1. NEAT1 binds HEXIM1 and is required for assembly of the HEXIM1–DNA-PK–paraspeckle components ribonucleoprotein complex (HDP-RNP), which contains DNA-PK subunits and multiple paraspeckle-associated proteins. The HDP-RNP interacts with cGAS and its partner PQBP1. Following exposure to foreign DNA, these interactions are remodeled, leading to release of paraspeckle proteins, recruitment of STING, and activation of DNAPKc and IRF3. Thus, the NEAT1–HEXIM1-containing HDP-RNP functions as a nuclear regulator of DNA-mediated innate immune signaling through the cGAS–STING pathway (104). Another notable example is MaIL1, which functions as a structural RNA component of the TLR4–TRIF signaling pathway. Following LPS stimulation, MaIL1 is induced and participates in the organization of the TLR4–TRIF signaling machinery. By interacting with and stabilizing optineurin (OPTN), MaIL1 facilitates downstream TBK1–IRF3 signaling and promotes type I interferon production (105). In this case, MaIL1 does not primarily regulate the abundance, localization, or intrinsic catalytic activity of a single signaling protein. Instead, MaIL1 functions as an integral RNA component of the TLR4–TRIF signaling machinery, stabilizes OPTN, and supports downstream TBK1–IRF3 signaling. Collectively, these studies demonstrate that lncRNAs can regulate innate immune signaling by controlling the assembly, stability, or disruption of protein complexes. Through scaffold formation, competitive interference, or structural incorporation into signaling platforms, lncRNAs can reshape the connectivity of signaling networks without necessarily changing the total abundance of their component proteins.

## 3 Discussion

The mechanisms reviewed above collectively support a hierarchical model of lncRNA-mediated protein regulation in innate immunity (**Figure 5**). At the molecular level, lncRNAs can regulate post-translational modifications, alter protein conformations, and facilitate or interfere with protein–protein interactions. These regulatory events are translated into changes in the functional state of target proteins, affecting their stability, subcellular localization, molecular activity, or ability to participate in signal-transducing protein complexes. The resulting changes in protein behavior can, in turn, reshape the magnitude, duration, and spatial organization of innate immune responses. Thus, rather than representing isolated molecular effects, lncRNA–protein interactions can be viewed as regulatory interfaces that connect specific molecular mechanisms to protein-level consequences and ultimately to immune outcomes.

**Figure 5:**
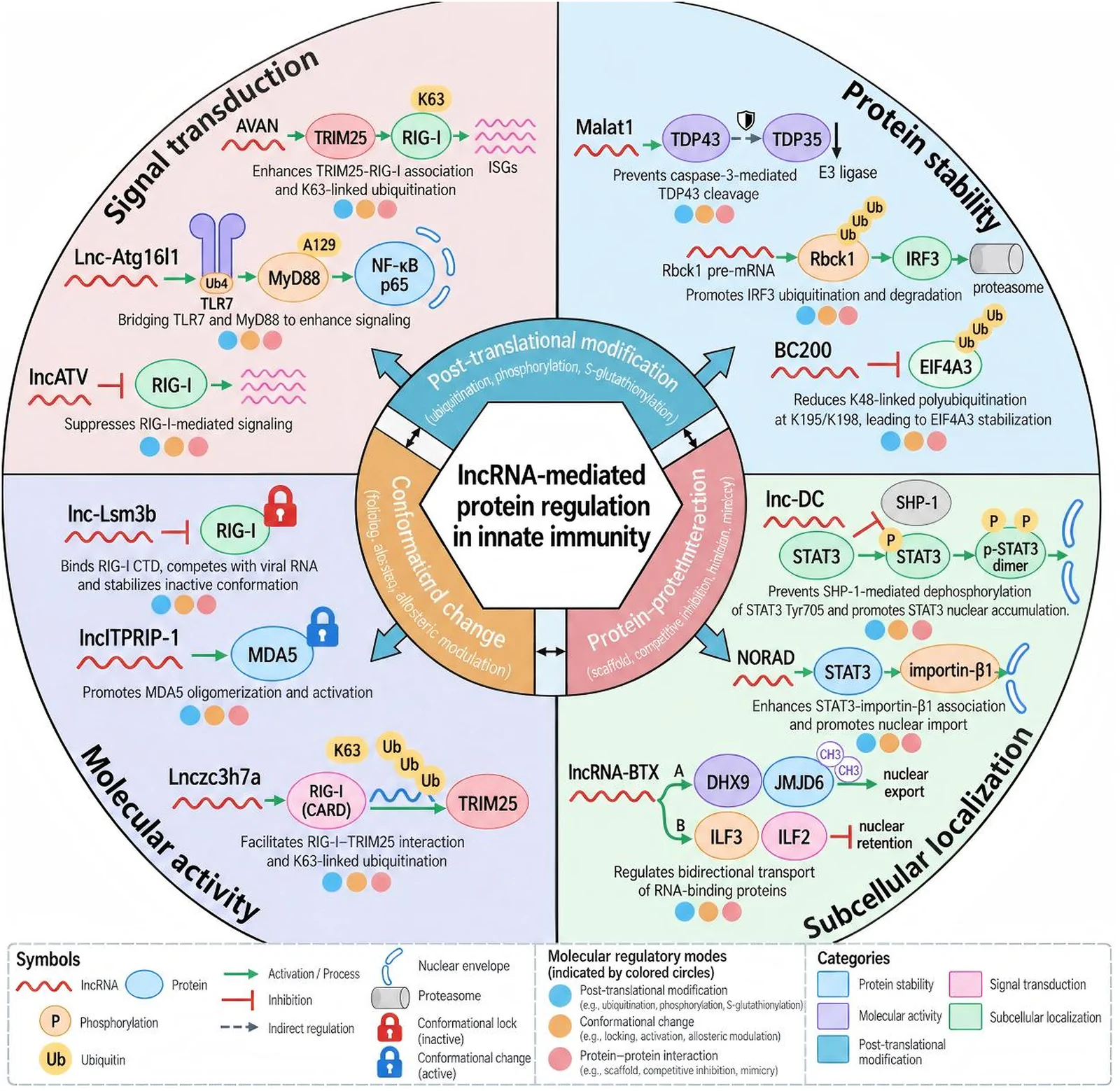
Integrated framework of lncRNA-mediated protein regulation in innate immunity. LncRNA–protein interactions regulate innate immunity through a hierarchical relationship between molecular regulatory mechanisms, protein-level consequences, and immune outcomes. At the molecular level, lncRNAs modulate post-translational modifications, protein conformations, and protein–protein interactions. These regulatory modes alter the stability, subcellular localization, molecular activity, and signal-transducing capacity of target proteins, thereby reshaping innate immune responses. Across these regulatory layers, lncRNA-mediated effects frequently converge on functionally important signaling nodes, can integrate multiple molecular mechanisms, and vary according to cellular and immunological context. Together, these features illustrate how lncRNAs translate specific molecular interactions into context-dependent changes in protein function and innate immune states.

Several recurrent principles emerge within this framework. First, lncRNA-mediated regulation is frequently concentrated at functionally important nodes within innate immune signaling networks, including pathogen sensors, signaling adaptors and kinases, and downstream transcription factors. Second, individual lncRNAs can integrate more than one molecular mechanism, allowing coordinated regulation of different properties of the same protein or multiple components within a signaling pathway. Third, the consequences of lncRNA–protein interactions are strongly dependent on cellular and immunological context, including infection status, cell type, and the surrounding signaling environment. Finally, some studies suggest that lncRNAs may regulate defined protein states or functional interfaces without necessarily altering total protein abundance, raising the possibility of functional selectivity. However, direct evidence for such selectivity remains limited and should not yet be considered a universal property of lncRNA-mediated regulation.

### 3.1 Recurring regulation at key innate immune signaling nodes

A recurring feature of the reported examples is the concentration of lncRNA-mediated regulation at functionally important nodes within innate immune signaling pathways. These nodes include pathogen sensors, adaptor proteins and kinases, and downstream transcription factors, where changes in protein activation, post-translational modification, localization, or protein–protein interactions can have substantial effects on downstream signal propagation. Rather than affecting signaling indiscriminately, lncRNAs can act at selected points within these pathways, thereby altering the strength or duration of the response. From this perspective, the regulatory nodes identified in the literature can be broadly viewed according to their position in signal initiation, transmission, and execution.

#### 3.1.1 Pattern recognition receptors: signaling initiation nodes

Pattern recognition receptors and related innate immune sensors define the earliest stage at which microbial or danger-associated signals are translated into intracellular signaling. At this level, regulation of receptor conformation, ligand accessibility, oligomerization, and receptor–adaptor engagement can alter the threshold for pathway activation without necessarily changing receptor abundance. The studies reviewed here indicate that lncRNAs can act at several of these control points, although the available mechanistic evidence is unevenly distributed across PRR families.

The clearest protein-centered examples currently involve cytosolic RNA sensing. RIG-I and MDA5 are regulated through changes in receptor conformation and oligomeric state, as illustrated by the opposing effects of lnc-Lsm3b and lncITPRIP-1 (73, 74, 106–108). TLR-associated regulation is also represented, particularly at the level of receptor–adaptor organization, whereas the newly incorporated inflammasome literature extends this principle to NLR- and AIM2-associated sensing complexes. In parallel, NEAT1-containing ribonucleoprotein complexes link lncRNA regulation to cytosolic DNA sensing through the cGAS–STING axis. By contrast, direct protein-centered regulation of CLR proteins remains poorly defined; available CLR-associated examples are largely downstream or indirect and therefore provide weaker evidence for direct receptor regulation.

Taken together, these observations suggest that lncRNAs can influence innate immune sensing at multiple entry points, but the current literature is strongly biased toward RLR-associated antiviral pathways. This imbalance should not be interpreted as evidence that lncRNA-mediated control is inherently restricted to RNA sensing. Rather, it highlights both the concentration of existing mechanistic work in a few well-studied systems and the need for comparable studies in less characterized PRR pathways.

#### 3.1.2 Adaptor proteins and kinases: signaling amplification nodes

Once a sensing receptor has been engaged, innate immune signals are transmitted through adaptor proteins, ubiquitin-dependent signaling assemblies, and kinases that determine whether the initial stimulus is amplified, sustained, or terminated. This intermediate layer is particularly sensitive to protein–protein interactions and post-translational modifications because signaling competence often depends on transient complex formation, higher-order assembly, and reversible changes in protein state rather than on protein abundance alone.

The studies reviewed in Section 2 show that lncRNAs can regulate this amplification layer at several mechanistic levels. In TLR pathways, lncRNAs can influence receptor–adaptor organization involving MyD88 or TRIF. In antiviral RNA sensing, regulation converges on TRIM25–RIG-I complexes, MAVS-associated signaling, and TBK1 activity (109, 110). DNA-sensing pathways similarly depend on the organization of cGAS–STING-associated complexes and downstream IRF3 activation. Across these systems, the immediate molecular events differ, but the functional consequence is similar: lncRNAs alter whether signaling intermediates assemble productively, acquire activating or inhibitory post-translational modifications, remain enzymatically competent, or undergo degradation, as illustrated by linc-AhRA-mediated control of TBK1 turnover (111).

This convergence identifies adaptor and kinase nodes as major points of signal control rather than passive intermediates between receptors and transcription factors. Because these proteins often integrate inputs from more than one receptor system, lncRNA mediated regulation at this level may influence several upstream pathways simultaneously. The available evidence therefore supports the view that signal amplification nodes provide an efficient point at which lncRNAs can adjust both the strength and duration of innate immune responses.

#### 3.1.3 Key transcription factors: signaling output nodes

The final outcome of innate immune signaling is determined by transcription factors that convert upstream activation into defined gene-expression programs. At this stage, regulation is no longer limited to whether a pathway is active or inactive; it also determines the magnitude, duration, and composition of the transcriptional response. IRF3, STAT family proteins, and NF-κB therefore function as major output nodes at which several protein-level regulatory mechanisms converge.

The studies reviewed here show that lncRNAs can influence these transcription factors through changes in phosphorylation state, nuclear localization, interaction with inhibitory or competing partners, and access to target promoters. The mechanisms involving lncRNA-ISIR, NORAD, and Lethe exemplify these distinct modes of control without requiring major changes in total transcription-factor abundance (61, 63, 91). Similar principles are seen in other lncRNA–protein interactions in which regulation of a defined protein state is sufficient to alter downstream gene expression.

This level of regulation is important because transcription factors integrate signals arriving from multiple upstream receptor systems. As a result, lncRNA-mediated control of a transcriptional output node can reshape responses initiated by different PRRs while preserving much of the upstream signaling architecture. Such regulation may therefore provide a means of adjusting inflammatory or antiviral gene expression with greater functional specificity than altering the abundance of entire signaling pathways. At the same time, the degree to which this selectivity is maintained across cell types and immune states remains incompletely resolved.

### 3.2 Integration of multiple regulatory mechanisms

The studies reviewed above indicate that lncRNA-mediated protein regulation often extends beyond a single molecular event. Instead, individual lncRNA–protein interactions can couple distinct regulatory layers, allowing an initial interaction to propagate through post-translational modification, protein localization, complex assembly, or protein stability. Several recurring patterns are evident. Malat1 exemplifies a sequential regulatory cascade in which an upstream RNA–protein interaction ultimately affects IRF3 stability (41), whereas lnc-DC links maintenance of STAT3 phosphorylation to its nuclear accumulation (60). lncRNA-BTX coordinates the spatial redistribution of multiple antiviral regulators through distinct protein interactions (66), while Lnczc3h7a couples signaling-complex assembly to TRIM25-mediated ubiquitination and activation of RIG-I (82).

These mechanisms are not equivalent: some represent sequential downstream consequences of a single initiating event, whereas others involve coordinated regulation of multiple proteins or molecular states. Their common feature is that the functional output of an lncRNA–protein interaction can extend beyond the immediate binding event and emerge from changes propagated across several regulatory layers. This distinction is important when interpreting multi-mechanism regulation, because downstream consequences should not be assumed to represent independently controlled functions unless the relevant causal steps have been experimentally demonstrated.

### 3.3 Immune state-dependent regulation

lncRNA-mediated protein regulation is also strongly conditioned by immune state. Infection or inflammatory stimulation can alter lncRNA abundance, localization, and the availability of interacting proteins, thereby changing when and to what extent a given lncRNA–protein interaction influences signaling. This context dependence is evident across several regulatory axes. Viral infection reduces Malat1 expression and thereby shifts the TDP43–TDP35–RBCK1 pathway toward increased IRF3 stability (41), whereas lnc-Lsm3b is induced later during RNA-virus infection and contributes to negative feedback on RIG-I signaling (73). Similarly, inflammatory induction of Lethe limits NF-κB output through RelA-dependent regulation (91). These examples illustrate that lncRNA-mediated effects can change with the timing and state of immune activation rather than functioning as constitutive regulatory inputs.

Immune-state dependence therefore adds a temporal and contextual dimension to the protein-centered mechanisms discussed in Section 2. The magnitude and direction of an lncRNA-mediated effect may depend on when the RNA is expressed, whether its protein target is available in the appropriate molecular state or compartment, and the cellular context in which the interaction occurs. Defining these variables will be important for distinguishing broadly conserved regulatory principles from mechanisms that operate only within particular cell types, stimuli, or stages of an immune response.

### 3.4 Potential functional selectivity of lncRNA-mediated protein regulation

A recurring feature of the studies discussed above is that lncRNAs can regulate defined molecular states or functional interfaces of immune proteins without necessarily altering their overall abundance. This distinction is potentially important because conventional genetic perturbations, such as gene knockout or knockdown, reduce the amount of the target protein and may consequently affect multiple functions associated with that protein. By contrast, direct lncRNA–protein interactions can act at specific regulatory sites, domains, or protein interfaces, providing a potential mechanism for modulating individual signaling events independently of changes in total protein abundance.

lnc-DC provides one of the clearest examples supporting this concept. lnc-DC interacts with STAT3 and prevents SHP-1-mediated dephosphorylation of Tyr705, thereby maintaining STAT3 in a phosphorylated state and promoting its downstream activity (60). Importantly, this regulatory effect is associated with control of STAT3 phosphorylation rather than a change in total STAT3 abundance. Thus, the lnc-DC–STAT3 interaction illustrates how an lncRNA can regulate a defined functional state of a signaling protein without relying on changes in protein expression.

NKILA illustrates a related principle at the level of regulatory-site accessibility. NKILA associates with the NF-κB/IκBα complex and masks phosphorylation sites in IκBα that are required for IKK-dependent phosphorylation and subsequent NF-κB activation (90). Rather than reducing the abundance of IκBα or NF-κB, NKILA alters the accessibility of a functionally important regulatory region. This mechanism therefore provides another example of how an lncRNA–protein interaction can modulate a specific molecular event within a signaling pathway. However, such mechanistic specificity should not be interpreted as evidence that other functions of the target proteins remain unaffected.

Several additional examples suggest that this principle may extend to other regulatory states, although the evidence for functional selectivity is less direct. lncRNA-ISIR interacts with the DNA-binding domain of IRF3 and facilitates events associated with TBK1-dependent IRF3 phosphorylation and activation (61), whereas lnc-Lsm3b binds RIG-I and stabilizes a conformational state that restricts receptor activation (73). These studies demonstrate that lncRNAs can engage defined protein domains or conformational states. However, they primarily establish mechanism-specific regulation and do not directly demonstrate that other functions of IRF3 or RIG-I remain unchanged. They should therefore be regarded as examples that are consistent with, rather than definitive evidence for, functional precision.

This distinction is particularly relevant in innate immune signaling, where individual proteins often participate in multiple molecular processes. Regulation through a defined phosphorylation site, protein domain, conformational state, or interaction interface could in principle provide greater functional selectivity than altering total protein abundance. The available studies therefore support the existence of a molecular basis through which lncRNA–protein interactions may achieve functional precision. Nevertheless, evidence that such regulation selectively modifies one protein function while preserving other physiological activities remains limited.

Functional precision should therefore be considered an emerging concept rather than an established or universal property of lncRNA-mediated protein regulation. Demonstrating this principle will require experiments that explicitly distinguish changes in protein abundance from changes in individual functional outputs and determine whether disruption of a specific lncRNA–protein interaction selectively affects one signaling function while preserving others. Structural characterization of RNA–protein interfaces, domain-specific mutagenesis, interaction-deficient mutants, and complementary loss-of-function and rescue experiments will be particularly important for establishing the molecular basis and biological extent of such selectivity. Selectivity may also arise from the RNA side of the interface because RNA secondary and tertiary structures can create context-dependent protein-binding surfaces whose accessibility changes with RNA folding and cellular state (112).

### 3.5 Limitations, emerging questions, and priorities for the field

Several limitations of the current evidence should be considered. First, the field remains dominated by mechanistic studies of individual lncRNA–protein pairs, often in a limited number of cell lines or infection models, and relatively few interactions have been validated across primary cells, animal models, and human disease samples. Second, the evidence base is heterogeneous: some examples reviewed here involve canonical innate immune sensors and signaling proteins, whereas others, such as NRON–NFAT and NEAT1–STAT6, are retained only as explicitly identified comparators that illustrate transferable principles of protein-centered immune regulation. These comparator examples should not be interpreted as direct evidence for canonical innate immune signaling. Third, direct RNA–protein binding, causal effects on a defined protein state, and downstream immune phenotypes are not demonstrated with equal depth in every study. Finally, several regulatory axes span more than one category in our framework, for example, lnc-DC couples phosphorylation to STAT3 nuclear accumulation, whereas Lnczc3h7a links complex assembly to RIG-I ubiquitination and activation. The four protein-level dimensions should therefore be viewed as interconnected organizing axes rather than mutually exclusive mechanistic classes. In such cases, we classify the regulatory axis according to the predominant protein-level consequence emphasized by the available evidence while explicitly acknowledging secondary effects across other dimensions. These limitations temper broad generalization and highlight the need for orthogonal interaction assays, endogenous perturbation, rescue experiments, temporal analysis, and validation in physiologically relevant models.

Mechanistic evidence should therefore be interpreted hierarchically. Detection of an association by pull-down, RIP, or related assays establishes enrichment or proximity but does not necessarily demonstrate direct binding or causal regulation. Stronger evidence is obtained when interaction mapping is complemented by purified-component binding assays, domain or nucleotide mapping, structure-guided mutagenesis, endogenous perturbation, interaction-deficient mutants, and functional rescue (113, 114).

A further unresolved issue is stoichiometry. Functional interpretation of an lncRNA-mediated interaction requires consideration of transcript copy number and, for protein-binding mechanisms, binding affinity, protein abundance, subcellular colocalization, and the fraction of each component engaged in the complex (114, 115). Low-abundance RNAs may still exert substantial local effects, but such mechanisms should be demonstrated rather than assumed. More broadly, nucleic acid–protein condensates can alter the timing and magnitude of innate immune signaling, although whether comparable mesoscale organization applies to most lncRNA–protein axes reviewed here remains unresolved (116).

An emerging question is whether lncRNAs can regulate multifunctional protein hubs that coordinate several innate immune pathways. TRIM29 is relevant in this context. Evidence from cancer studies indicates that TRIM29 expression is subject to regulation by non-coding RNA networks, including lncRNA-associated regulatory pathways (117). Independently, TRIM29 has been shown to regulate multiple components of innate immune signaling. TRIM29 promotes the ubiquitination and degradation of NEMO, STING, and MAVS, thereby modulating type I interferon and NF-κB responses (118–120). It also interacts with PERK and promotes its SUMOylation and stability during viral myocarditis (121), while more recent work shows that TRIM29 promotes K48-linked ubiquitination and degradation of NLRP6 and NLRP9b, thereby restraining IFN-λ production and inflammasome activation during enteric RNA virus infection (122). Together, these observations raise the possibility that lncRNA-dependent regulation of TRIM29 expression or activity could influence multiple innate immune pathways. Direct mechanistic evidence for this relationship in innate immune settings remains limited, and determining whether lncRNAs regulate TRIM29 abundance, post-translational state, or protein interactions during infection or inflammation represents an important question for future studies.

Two additional issues are becoming increasingly important. Recent work on the IAV-associated lncRNA IPAN shows that host lncRNAs can also protect viral proteins from RIG-I/TRIM25-dependent degradation, emphasizing that protein-stability control can be exploited by viruses as well as by host defense (123). In parallel, evidence that transcripts annotated as lncRNAs can encode functional micropeptides—including a recently described MALAT1-derived micropeptide that modulates influenza A virus replication—complicates a strict coding versus non-coding classification (124–126). Future studies should therefore establish whether a phenotype is mediated by the RNA molecule itself, by an encoded peptide, or by both, particularly when assigning mechanism to disease-associated lncRNA loci.

## 4 Conclusions and future perspectives

The evidence reviewed here supports an integrated view in which lncRNAs fine-tune innate immunity through direct or indirect regulation of protein function. At the mechanistic level, lncRNA–protein interactions can modulate post-translational modifications, alter protein conformations, and facilitate or interfere with protein–protein interactions. These molecular regulatory modes, in turn, influence distinct but interconnected properties of target proteins, including their stability, subcellular localization, molecular activity, and capacity to transmit immune signals. Rather than representing independent regulatory events, these mechanisms can converge at key signaling nodes to shape the magnitude, duration, and spatial organization of innate immune responses. Future studies should define how these regulatory modes are coordinated under physiological and pathological conditions and determine whether specific lncRNA–protein interfaces can be selectively targeted to modulate innate immunity without broadly disrupting the functions of their associated proteins.

Therapeutically, these mechanisms suggest several levels of intervention. Disease-promoting lncRNAs can in principle be reduced with antisense oligonucleotides, siRNA-based approaches, or other programmable RNA-targeting strategies, although subcellular localization, delivery, durability, and off-target effects remain major constraints (127–130). A more mechanism-selective strategy would be to disrupt or stabilize a defined lncRNA–protein interface without eliminating the entire RNA or protein. Small molecules that recognize RNA structures or pockets formed at RNA–protein interfaces provide emerging proof of principle, but application to innate-immune lncRNA complexes remains largely preclinical (131–133).

Although clinical evidence remains limited, emerging studies have begun to define specific tumor contexts and biomarker-selected patient populations that may be suitable for future lncRNA-directed therapies. Available evidence also provides some indication of the cancer types and patient populations that may be most suitable for future lncRNA-directed therapies. At present, the strongest rationale comes from tumors in which the target lncRNA is highly or lineage-selectively expressed and is functionally required for tumor maintenance or progression. Melanoma provides a particularly clear example. SAMMSON is detectable in more than 90% of human melanomas, and its depletion markedly reduces melanoma-cell viability irrespective of BRAF, NRAS, or TP53 mutational status. Moreover, SAMMSON targeting sensitized melanoma to MAPK-pathway inhibitors in patient-derived xenograft models, suggesting that SAMMSON-positive melanoma, including tumors treated with MAPK-directed therapy, represents a plausible candidate population for future lncRNA-targeted intervention (134).

A second potential population comprises patients with MALAT1-high, metastasis-prone lung cancer. MALAT1 expression is associated with the metastatic phenotype of lung cancer cells, and antisense oligonucleotide-mediated MALAT1 inhibition reduced metastatic colonization in xenograft models. These data provide a preclinical rationale for evaluating MALAT1-directed therapy in patients whose tumors show high MALAT1 expression and a high risk of metastatic progression, although clinical efficacy in this population has not yet been established (135).

More direct clinical evidence for biomarker-based patient selection comes from H19-regulated therapeutic strategies. BC-819 (H19-DTA), which uses H19 regulatory sequences to drive tumor-selective diphtheria-toxin expression rather than directly silencing H19 RNA, has been evaluated specifically in H19-positive tumors. Clinical studies have included patients with recurrent or multifocal non-muscle-invasive bladder cancer, including cases that had failed prior intravesical therapy, as well as patients with H19-positive recurrent ovarian or primary peritoneal cancer, particularly platinum-resistant or platinum-refractory disease (136, 137). These studies illustrate how lncRNA-associated biomarkers may be used prospectively to define patient populations for targeted treatment.

By contrast, evidence for other tumor types remains more exploratory. In a phase I study of the antisense oligonucleotide Andes-1537 in heavily pretreated advanced solid tumors, prolonged stable disease was observed in individual patients with pancreatic cancer and cholangiocarcinoma, but no partial or complete responses were reported (138). These observations are insufficient to identify pancreatic or biliary tract cancers as established responsive populations and instead highlight the need for larger, biomarker-guided studies.

Taken together, current evidence suggests that the patients most likely to benefit from future lncRNA-directed strategies are not defined by histology alone. More appropriate candidates are likely to be those whose tumors show high or lineage-restricted expression of the target lncRNA, functional dependence on the corresponding lncRNA or lncRNA–protein regulatory axis, and adequate accessibility of the target tissue to the therapeutic modality. Prospective measurement of target RNA abundance, confirmation of the relevant lncRNA–protein interaction or downstream dependency in tumor tissue, and pharmacodynamic evidence of target engagement will therefore be essential for patient selection in future clinical trials.
